# The prevalence and functional impact of musculoskeletal conditions amongst clients of a primary health care facility in an under-resourced area of Cape Town

**DOI:** 10.1186/1471-2474-11-2

**Published:** 2010-01-04

**Authors:** Romy Parker, Jennifer Jelsma

**Affiliations:** 1 School of Health and Rehabilitation Sciences, University of Cape Town, Cape Town, South Africa

## Abstract

**Background:**

The extent of disease burden of musculoskeletal conditions (MSC) not due to injury has not been well determined in sub-Saharan Africa. The 1999 Global Burden of Disease study estimated the prevalence of osteoarthritis and rheumatoid arthritis to be 150/100,000 compared to 1,500/100,000 in Europe. The objective of the study was to determine the prevalence of MSC and the functional implications in a sample of people attending community health centres in Cape Town, South Africa.

**Methods:**

A cross-sectional, descriptive study was conducted in clinics in two resource poor communities. Phase I consisted of screening and those who screened positive for peripheral or spinal joint pain went on to complete Phase II, which included the Stanford Health Assessment Questionnaire.

**Results:**

1005 people were screened in Phase I. Of these, 362 (36%) reported MSC not due to injury in the past three months. Those with MSC had higher rates of co-morbidities in every category than those without. The mean Disability Index for those with MSC was mild to moderate and moderate to severe in those over 55 years.

**Conclusions:**

Although the sample may not be representative of the general community, the prevalence is considerably greater than those reported elsewhere even when the population of the catchment area is used as a denominator, (367/100 000). The common presentation of MSC with co-morbid diabetes and hypertension requires holistic management by appropriately trained health care practitioners. Any new determination of burden of disease due to MSC should recognise that these disorders may be more prevalent in developing countries than previously estimated.

## Background

Musculoskeletal conditions (MSC) are prevalent in developed countries and are commonly reported to result in pain, disability and loss of function [1-3]. The burden of disease (BoD) methodology has been used to quantify the impact of these conditions and it reports the burden in terms of Disability Adjusted Life Years (DALYs), which reflect both the mortality (years of life lost, YLLs) and morbidity (years of life lived with disability, YLDs) associated with a condition. Correct estimates of DALYs require accurate prevalence data and an estimation of the impact of the disability associated with a condition, a disability weight. Currently there are plans to estimate the global burden of disease based on 2005 data (Joshua Solomon, personal communication) and it is essential that data be available upon which to base accurate estimates of the burden due to non-fatal conditions in general and musculoskeletal conditions in particular. An accurate estimation will assist policy makers in making well informed decisions related to resource allocations and subsequent management of those with MSC.

In the 1990 Global BoD study, MSC were estimated to account for 4.3% of the DALYs and osteoarthritis (OA) was the third largest contributor (27.3%) to the YLDs in developed countries. In contrast, MSC contributed only 1% to DALYs and OA was not listed amongst the top ten major contributors to YLDs in the developing world[4]. This discrepancy relates to the estimated all age prevalence rates of 325 for Rheumatoid Arthritis (RA) and 1 161/100 000 for OA in Europe compared to 34 and 110/100 000 in Sub-Saharan Africa. In the 2000 South African Burden of disease study[5], MSC were estimated to contribute relatively little to the burden of all conditions examined and were ranked 20^th ^out of 24 conditions. However, this burden was not calculated from empirical data but was based on the ratios of YLDs to YLLs estimated by the World Health Organisation (WHO) for the Afro E region, of which South Africa is a part, for each of the disease categories[5]. It is clear that if the burden estimates mentioned above are accepted, the resources diverted to the management of musculoskeletal conditions will be relatively few. It becomes necessary to therefore examine to what extent these estimates might be correct.

There appear to be very few studies in developing countries that present grounds for challenging the above prevalence estimates. The MSC are commonly overlooked by researchers due to the apparent greater urgency of investigating infectious diseases[6]. The WHO has recognized that: "much research has been directed at fatal diseases, whereas crippling diseases are commonly neglected - yet the social and economic burden which the latter impose, is probably greater"[7].

There is strong evidence to show the relationship between disability and MSC with indications that people living in socially deprived areas are more prone to musculoskeletal symptoms and have poorer resultant functional outcomes[3,8]. A study on the burden of disease in Zimbabwe indicated that OA was the eleventh highest contributor to YLDs[9]. In addition, a community based survey in a Zimbabwe township indicated that OA and back pain were the second and third most common causes of disability with a prevalence rate of 1531/100 000 for back disorders and OA combined[10], a rate much closer to the European prevalence rate reported above than the BoD estimates.

There is a paucity of information relating to the prevalence of MSC in South Africa and the functional impact of these conditions. A few publications have reported on the epidemiology of RA in specific ethnic groups in South Africa but do not report on the prevalence of MSC[2,8,11-13]. Unfortunately it was beyond the scope of this present study to undertake a community based prevalence study. However, as the prevalence of joint and muscle conditions in those who utilise public facilities should influence the resources that should be made available for the management of these conditions, it was deemed useful to establish the prevalence of these conditions in adult attendees of clinics in under-resourced areas. The aim of this study was to determine the prevalence of MSC, defined as those presenting with either peripheral or spinal joint pain, and their functional implications in a sample of people attending two community health centres in Cape Town, South Africa.

## Methods

### Research setting

A cross-sectional, descriptive study was conducted in clinics serving two resource poor communities each of approximately 50 000 inhabitants which were historically disadvantaged in the apartheid era[14]. The population served by these clinics are approximately equivalent in terms of gender and ethnic groups and the majority of the population are between the ages of 18 - 34 years[14]. The most common languages spoken were IsiXhosa (45%) and Afrikaans (41%). In the one area 53% of residents live in informal housing/shacks with one third having no access to electricity and indoor running water. In the other community, 71% live in formal housing with both electricity and running water. Unemployment in both suburbs is high (one-third to one-half unemployed) and less than 10% of the population of either suburb has completed all 12 years of schooling[14].

The clinics at which data collection took place were chosen for the study as they are well established facilities with a good base of multidisciplinary team members. The clinics are easily accessible being within walking distance of all residents. Both clinics are well attended, serving 600, and 400 people per day respectively.

### Sample

A sample of convenience was obtained with all adults attending the health care centres on the days when data collection took place being eligible for inclusion. Inclusion criteria included being over the age of 18, willing to participate in the study, and attending the clinic for personal health-related reasons. Subjects too ill to participate, or who could not answer the questionnaire in one of the three languages of English, Afrikaans or isiXhosa were excluded. In order to detect a prevalence rate of 14% (12-16% confidence intervals) it was calculated that a sample of 791 would be required[15].

### Measurement instruments

A screening instrument was developed which included Phase I and Phase II of the COPCORD questionnaire and the Stanford Health Assessment Questionnaire - Disability Index (HAQ).

The COPCORD is a valid and reliable measurement instrument which has been used in many developing communities[16,17]. Phase I of the COPCORD was modified to include South African language groups (English, Afrikaans, IsiXhosa, IsiZulu and Other). Subjects, who screened positive for peripheral or spinal joint pain (PJP/SJP) not due to trauma or injury on Phase I of the COPCORD, entered the second phase of the study, completing the Phase II COPCORD and the HAQ.

A valid, reliable and culturally appropriate version of the HAQ in South African English and Afrikaans was used[18]. In the HAQ, the Disability Index (DI) is calculated based on the categories of dressing, arising, eating, walking, hygiene, reach, grip, and common activities and can range from 0 (no disability) to 3 (Unable to perform in any category).

The questionnaires were translated into isiXhosa and Afrikaans where translated versions were not available, using a forward-translation, back-translation methodology. Following piloting with 10 subjects, the method of administration of the questionnaires was changed from self-administration to administration by research assistants to prevent omission of data.

### Procedure

Ethical approval for the study was granted by the Medical Research Ethics Committee of the University of Cape Town. Permission to conduct the study was granted by the clinic authorities. Subjects were approached while waiting in queues for medical attention. Each subject completed a written informed consent prior to Phase I. Subjects reporting PJP/SJP not due to injury were asked to proceed to Phase II of the study.

### Statistical Analysis

This study made use of descriptive statistical analysis to determine mean values and frequencies of demographic information gathered. The point prevalence estimate of people affected by musculoskeletal conditions in the given sample was calculated. Sub-group analysis of the DI scores were performed using frequency tables, t-tests, the one-way ANOVA and Spearman correlations.

## Results

### Total Sample

There were 1290 people approached to participate in the study, 266 did not wish to participate and 19 were not eligible. Thus a total of 1005 people were screened (the population). Of these, 362 reported PJP/SJP not due to injury in the past three months (the cases).

Females represented 63% of the population. The average age of the population was 46 ± 16 years (range 18 - 97); with the 40 to 60 year old age group representing 40% of the population. Almost two thirds (63.1%) were not married and the majority (73.6%) lived in brick housing. Almost all were literate (91.8%) with 41% obtaining school leaving certificates. Previous or current smoking habits were reported by 52% and 39% of the population reported current or previous alcohol consumption.

The majority of the population (64%) were visiting the day hospital to consult with a nurse or doctor. The most common health problems experienced by participants visiting the clinic in the last three months were hypertension; joint/muscle pain; diabetes; lung; stomach and heart problems (Table 1). The number of subjects attending the clinic for musculoskeletal problems *only*, which were not due to trauma or injury, was 70 (6.9%).

**Table 1:** The most common health problems experienced by all participants (n = 1005), the controls (n = 643) and the cases, those with MSC (n = 362).

	% Problems experienced in the last three months in the controls (n = 643)	% Problems experienced in the last three months in cases (those with MSC) (n = 362)	% Problems experienced in the last three months by all participants (n = 1005)
Joint/muscle pain	0	100	36.0
Hypertension	21.4	59.1	35.1
Diabetes	15.7	24.8	19.1
Lung Problems	12.8	16.1	14.2
Stomach Problems	4.2	16.1	10.0
Heart problems	6.6	18.9	11.0
Depression	3.6	13.0	6.9
Infection	4.1	3.9	3.9

### Comparison of Cases and Controls

Three hundred and sixty two subjects reported PJP/SJP that was not due to trauma and progressed to Phase II. This represents a prevalence rate of 36% in the clinic population. Table 2 compares the gender and housing conditions of the cases and the controls. Gender was associated with inclusion in the sample with more females belonging to this group.

**Table 2:** Comparison of controls and cases.

	Controls (n = 643) Frequency (%)	Cases (n = 362) Frequency (%)	Chi Square	P value
Gender				
Male	298 (46.4)	72 (19.9)	69.7	*< .001*
Female	345 (53.7)	290 (80.1)		
**Total**	**643**	**362**		
Housing				
Informal	179 (27.8)	85 (23.6)	2.2	0.138
Brick	464 (72.2)	276 (76.5)		

**Total**	**643**	**361**		

The mean age of the sample was 51.7 ± 15.3 yrs which was 8.5 years greater than the population mean (p < 0.001). The most common co-morbidities in the sample were hypertension (59.1%), diabetes (24.8%) and heart problems (18.9%). As can be seen in Table 1; with the exception of infection, the cases reported more problems than the controls in every category of co-morbidity.

Back pain was by far the most common complaint, both in isolation (18.5%) and combined with peripheral pain (55%). In general, the majority of cases had pain in more than one joint, although as can be seen in Table 3, isolated back and knee pain were common.

**Table 3:** Involved joints reported by the cases (n = 362).

Joints	Frequency	%
Spine	67	18.5
Spine/hips	12	3.3
Spine/hips/knees	134	37
Spine/knees	56	15.5
Hips/knees	14	3.9
Hips	3	0.8
Knees	48	13.3
Other joints	28	7.7
Total	362	100

Pain was most commonly reported on awakening. With regard to stiffness, 80% (203) of the cases experienced stiffness after awakening or long periods of rest with 85% reporting improvement with movement or exercise. The stiffness was reported to last for more than 30 minutes by 51% (103) of this group. There was no relationship between anatomical region of symptoms and behaviour of pain or stiffness.

In the cases where a previous diagnosis had been made (15%), the majority (60%) had been diagnosed with joint pathology. The most common diagnosis was arthritis (76%) followed by OA (10%) and RA (5%).

### Functional Outcomes

**Figure 1:**
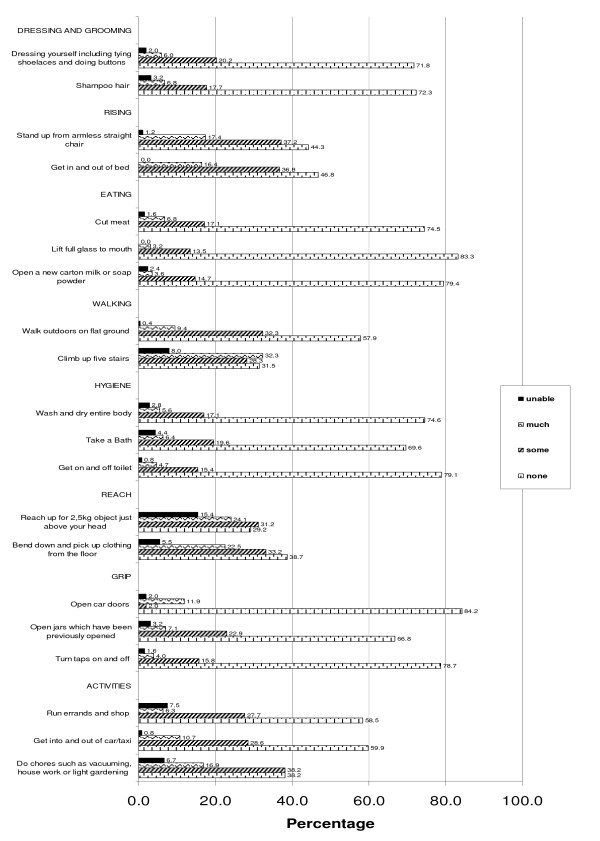
**Functional Outcomes on the HAQ of those presenting with PJP/SJP (the sample)**.

Figure 1 is a representation of the functional results as recorded with the HAQ. The categories of "Reaching", "Walking", "Rising" and "Activities" caused most difficulty for the greatest number of cases. The majority had at least "some difficulty" in *climbing up 5 stairs*; 15.4% of cases were "unable" to "*reaching up for a 2.5 kg object*. The categories of "Dressing and Grooming", "Eating", "Hygiene" and "Grip" caused the least functional limitations.

The mean DI for the sample was 0.8 (mild to moderate disability). The mean DI for males and females were 0.66 and 0.85 respectively. No significant difference was found between the two (p = 0.657, SD = 0.36). Exploration of the DI according to age revealed that mean DI increased with age with a positive correlation between age and DI (r = 0.31; p < 0.001).

## Discussion

The researchers were constrained in their choice of research design and undoubtedly a community based survey would have yielded more useful information. However the study did uncover some important results.

Over one third of clinic attendees had MSC not due to trauma or previous injury. This figure is higher than those reported in community based studies in the USA (24%), Mexico (17%) and the Philippines (16%)[7,16,19]. It is notable that the average age of the clinic attendees was 8.5 years older than the background population and it may be that an increase in MSC could be expected in an older population.

Using the figure of 362 people reporting MSC the community prevalence rate can be estimated. The catchment area of the clinics has a population of approximately 100 000, the community prevalence rate is therefore *at least *362/100 000 which is over twice the estimated BoD prevalence rate for sub-Saharan Africa of 144/100 000 for OA and RA combined[4]. It would appear that the prevalence of MSC affecting muscles and joints needs to be revisited in the next round of BoD estimations.

Although other studies have demonstrated that females are more likely to utilise public health services[1,20-22], the gender distribution in clinic attendees was approximately equal with 54% female. This was not so for subjects in the cases (those with PJP/SJP not due to injury) and as reported in the literature, the presence of joint and muscle pain was significantly associated with gender. More than 80% of the cases were female, a far larger proportion than a previous study which reported the ratio of women to men who were suffering from joint pain to be 1,6:1[7]. This increased prevalence of MSC in women, is a pattern recognised in many chronic musculoskeletal conditions including RA, OA and fibromyalgia[19,23].

The large number of patients attending the clinic either solely to have joint or muscle pain treated (7%) or with pain as a co-morbidity to their other illnesses (33%) implies that clinic staff need to be prepared to manage MSC well through accurate diagnosis and appropriate management. Staff need to be trained to recognise and refer patients to appropriate services. Such services range from tertiary care for surgical intervention to primary care rehabilitation services in the community.

Only 15% of the cases had been previously diagnosed and the majority of these with generic 'arthritis'. The majority had co-morbidities related to age and life-style, including chronic diseases such as hypertension, diabetes and heart problems. It would appear that management of the chronic diseases of life-style should include aspects of joint pain management and that a comprehensive management package should include medication, exercise and advice for all related co-morbidities. In addition, the link between depression (reported by 13% with joint pain) and joint pain needs attention. Previous studies have linked depression to RA and suggest this association to stem from the functional limitations experienced by people living with RA[24].

The back, knees and shoulders were the most commonly affected joints overall with slightly fewer males affected in each of these three groups. These findings are similar to those in other countries[7,25]. The mean DI for those with MSC was 0.8, indicating mild to moderate disability. This is a similar result to a previous study investigating joint disease in an outpatient clinic in Finland which reported more than half the population under investigation to have a DI between 0 and 1[26]. This is a somewhat surprising finding as previous authors have reported on poorer functional outcomes in people with MSC living in socially deprived areas[8]. A more comprehensive community based study would provide further insight.

In this study the 55+ age group had a mean DI indicating moderate to severe disability. It is recognised that MSC increase in prevalence and severity with age[20]. This is also reflected in the age distribution of the sample as the 55+age range made up 53% of the sample.

Whole body functions such as "running errands" were the most difficult for subjects. Subjects with combined joint involvement had the highest mean DI indicating a link between the number of joints involved and resultant disability. This suggest that people suffering from MSC might benefit from treatment programmes which focus more on rehabilitation and maintenance of gross motor and whole body functions.

Several limitations were identified in this study. Firstly the recruitment of subjects at health care centres may bias the results as this group may represent the less healthy members of their communities and as such the results should not be interpreted to reflect the prevalence of MSC in the community as a whole. During data collection, some subjects were lost due to the length of Phase II of the questionnaire with subjects being unable to complete interviews prior to leaving the queue for their appointment.

It is, therefore, recommended that future studies conduct door to door community surveys in order to determine a more accurate prevalence of rheumatic and musculoskeletal conditions. In addition the use of Phase III of the COPCORD study would greatly enhance information gained by making definite diagnoses and therefore determining an accurate prevalence.

## Conclusion

The prevalence of musculoskeletal conditions in this clinic based study was found to be 36%. Although the sample may not be representative of the community in general, the prevalence, even when the population of the catchment area is used as a denominator, is considerably greater than those reported in community studies in the developed and developing countries throughout the world.

There was an expected correlation between age and disability. Whole body functions described on the HAQ such as "running errands" caused considerable difficulty for subjects. This is also reflected in the finding that combined joint involvement causes more disability than isolated joint involvement. However, the extent of the disability was regarded as mild to moderate in the whole sample and moderate to severe in the older age group. These findings might have implications for establishing appropriate disability weights for QALY or DALY calculation.

When planning and providing appropriate services in primary care facilities, it is suggested that assessment of the community should be the first step taken[27]. The high prevalence rates of MSC in this sample group and the subsequent mild to moderate disability ensuing reinforces the previously highlighted need for appropriately trained health care practitioners at primary healthcare centres[2]. In developing countries such as South Africa where access to tertiary healthcare is limited, management of both the illness and rehabilitation of disability at these primary health care centres needs to be addressed.

## Competing interests

The authors declare that they have no competing interests.

## Authors' contributions

RP and JJ contributed to the study design, implementation, analysis and drafting of the manuscript. Both authors' read and approved the final manuscript.

## Pre-publication history

The pre-publication history for this paper can be accessed here:

http://www.biomedcentral.com/1471-2474/11/2/prepub
